# Morpho-Molecular Evidence Reveals Four Novel Species of *Gymnopus* (Agaricales, Omphalotaceae) from China

**DOI:** 10.3390/jof8040398

**Published:** 2022-04-13

**Authors:** Ji-Peng Li, Mei-Chen Pan, Yi Li, Chun-Ying Deng, Xiao-Min Wang, Bang-Xi Zhang, Chang-Tian Li, Yu Li

**Affiliations:** 1 Engineering Research Center of Chinese Ministry of Education for Edible and Medicinal Fungi, Jilin Agricultural University, Changchun 130118, China; lijipengfungi@163.com (J.-P.L.); panmc718@163.com (M.-C.P.); 2 College of Food Science and Engineering, Yangzhou University, Yangzhou 225127, China; liyi062@yzu.edu.cn; 3 Guizhou Institute of Biology, Guizhou Academy of Sciences, Guiyang 550009, China; chunyingdeng2022@163.com; 4 Soil and Fertilizer Research Institute, Guizhou Academy of Agricultural Sciences, Guiyang 550006, China; rdl1916@163.com (X.-M.W.); zbx@cau.edu.cn (B.-X.Z.)

**Keywords:** fungal taxonomy, Holarctic regions, morphology, phylogeny, saprotrophic fungi, sect. *Androsacei*, sect. *Impudicae*, sect. *Levipedes*

## Abstract

Nine collections of gymnopoid fungi were studied based on morpho-molecular characteristics. The macromorphology was made according to the photograph of fresh basidiomata and field notes, while the micromorphology was examined via an optical microscope. Simultaneously, the phylogenetic analyses were performed by maximum likelihood and Bayesian inference methods based on a combined dataset of nrITS1-nr5.8S-nrITS2-nrLSU sequences. Integrated analysis of these results was therefore, *G. efibulatus* belonging to sect. *Androsacei*, *G. iodes* and *G. sinopolyphyllus* belonging to sect. *Impudicae* and *G. strigosipes* belonging to sect. *Levipedes* are proposed as new to science. The detailed descriptions, colour photos of basidiomata and line-drawings of microscopic structures are provided. The comparisons with closely related species and a key to known species of *Gymnopus* s. str. reported with morpho-molecular evidence in China is also given.

## 1. Introduction

*Gymnopus* (Pers.) Roussel is a group of white-spored macrofungi with collybioid, rarely tricholomatoid, marasmioid or omphalinoid habit and distributed worldwide [1,2,3,4,5,6,7,8,9,10,11,12,13,14,15,16,17,18,19,20,21,22]. Its habit, causing the similarity in macromorphology, is the main reason for the confusion in taxonomy in the past. When revisiting the history of the genus, it is clear that the materials from Holarctic regions, comprised of Palaearctic and Nearctic subregions, were well studied [1,2,3,4,5,6,7,8,23,24]. Morphological studies on samples from Europe and northern Africa, a part of the Palaearctic subregion, and a few samples from North America, belonging to the Nearctic subregion, dominated the major revision of the generic concept [1,2,23,24,25]. However, the highly phenotypic plasticity that causes the close species often forms a complex, such as the *G. dryophilus* complex, making classification in the species level hard if only depends on morphology [26,27]. The taxonomic study was improved following the use of the polyphasic method. Studies on mating systems and isozymes were used to distinguish the *G. dryophilus* complex, confirming the morphological results [26,27]. The molecular–phylogenetic analysis plays a critical role in species recognition and generic revision as a tool for evolutionary inference. Based on the morphological study and multi-gene phylogeny, the concepts of *G. dryophilus* (Bull.) Murrill and *G*. *ocior* (Pers.) Antonín and Noordel. were clarified [4]. Additionally, several results inferred from this method inspired mycologists to reconsider the inter- and infrageneric taxonomic position [7,28,29]. Therewith, *G*. sect. *Androsacei* (Kühner) Antonín and Noordel. was proposed [30]. *Gymnopus* sect. *Vestipedes* subsect. *Impudicae* and subsect. *Vestipedes* were raised, representing two distinct sections now [2]. In addition to that, *G*. sect. *Vestipedes* (Fr.) Antonín, Halling and Noordel. was transferred to *Marasmiellus* Murrill but is currently called *Collybiopsis* Earle according to the nomenclature argument [31,32]. Lately, the monotypic section, *G*. sect. *Gymnopus*, was revised by including two additional small-sized species based on the polyphasic method of combined morpho-molecular evidence [18].

Currently, the study within the additional component of the Palaearctic subregion, the Asian north of the Himalayan mountains, is less covered that only 15 new taxa within *Gymnopus* sensu stricto (s. str.) were described therein [10,11,12,13,33]. Emending the type section of *Gymnopus* implied the importance of Chinese materials [18]. The reports regarding the Chinese individual(s) of *Gymnopus* s str. were at least beginning in 1892; Karsten reported a collection from Gansu Province, China, representing *Marasmius dryophilus* (Bull.) P. Karst. (≡*Gymnopus dryophilus* (Bull.) Murrill; author’s name printed as (Bolt.) Karst. by typographical errors) [34]. However, only 15 species were reported based on morpho-molecular evidence in China to date [16,17,18,33], which reveals that the knowledge of the genus is still deprived in this region. In this study, four new species of *Gymnopus* from China, including one Holarctic taxon, were proposed based on the polyphasic method, viz. combined macro- and micro-morphology and multi-gene (nrITS1-nr5.8S-nrITS2-nrLSU) phylogenetic analyses. Colour photos of basidiomata and line-drawings of their microstructures are present. Discussions about phylogenetic relationships, comparisons with close species and a key to known species within *Gymnopus* s. str. reported by morpho-molecular evidence, are also given.

## 2. Materials and Methods

### 2.1. Abbreviations for Specific Names and Additional Mycological Glossary

*G*. = *Gymnopus*; *Ma*. = *Marasmius*; *My*. = *Mycetinis*; *Pa*. = *Paragymnopus*; nom. prov. = nomen provisorium; s. str. = sensu stricto.

### 2.2. Specimen

Nine new collections of gymnopoid fungi from China were included in this study. Samples were photographed in the field and dried by a portable drier operated at 45 °C. The specimens were deposited in HMJAU (Herbarium of Mycology, Jilin Agricultural University, Changchun, China), HGAS-MF (Herbarium of Guizhou Academy of Sciences, Guiyang, China) and HMAS (Fungarium of the Institute of Microbiology, Chinese Academy of Sciences, Beijing, China), respectively. The herbarium abbreviations follow Index Herbariorum [35].

### 2.3. Morphological Studies

Macromorphological features were described based on photographs of fresh basidiomata and field notes. The colours terminology and code follow Kornerup and Wanscher [36]. Two types of lamellae were counted, of which the number of full-length lamellae is represented by ‘L’, and the number of lamellulae tiers is represented by ‘l’.

Tiny tissue was cut from the dried basidiomata using a sharp blade and then mounted in 5% KOH on a glass slide for microscopic observation via a light microscope (ZEISS Axioscope 5). When needed, the Congo Red solution was used to highlight the outline of microscopic structures, and Melzer’s reagent was used to test amyloid or dextrinoid reactions. In microscopic description, ‘n’ refers to the number of measured elements. Measurements of basidiospores are given as (a)b–c(d), of which ‘b–c’ refers to the minimum of 90% of the measured values. The main body excluding sterigmata or excrescences of basidia, basidioles and cystidia were measured.

### 2.4. DNA Extraction, Amplification and Sequencing

The dried tissue was used for genomic DNA extraction using NuClean Plant Genomic DNA Kit (Cowin Biotech Co., Ltd., Taizhou, China). The nuclear ribosomal internal transcribed spacer (nrITS) and nuclear ribosomal large subunit (nrLSU) sequences were amplified using primer pairs of ITS5/ITS4 and LR0R/LR5, respectively [37,38,39]. The polymerase chain reaction (PCR) programs were followed according to Li et al. [18], and the PCR products were sent to Sangon Biotech (Changchun, Jilin, China) for sequencing.

### 2.5. Phylogenetic Analyses

Sequences generated in this study were combined with those obtained from GenBank by Basic Local Alignment Search Tool (BLAST) and then added to the matrix used by Li et al. [18], which covered all the sections of *Gymnopus* s. str. Based on two overlapping reads, targeted sequences were assembled and trimmed via BioEdit v.7.0.9 [40]. Quality control, such as degenerate bases checking, was completed before depositing the sequences to be used in GenBank (see Table 1) [41]. The ML (Maximum Likelihood) and BI (Bayesian Inference) analyses follow Li et al. [18]. The Bootstrap Proportions ≥70% for ML analysis (ML-BP) and Posterior Probability ≥0.95 for Bayesian Inference analysis (BI-PP) were considered a significant value. The alignment file of the combined dataset used for phylogenetic analyses and both the two phylograms generated from two methods was deposited in Treebase (https://www.treebase.org/treebase-web/home.html (accessed on 18 March 2022).

## 3. Results

**Figure 1:**
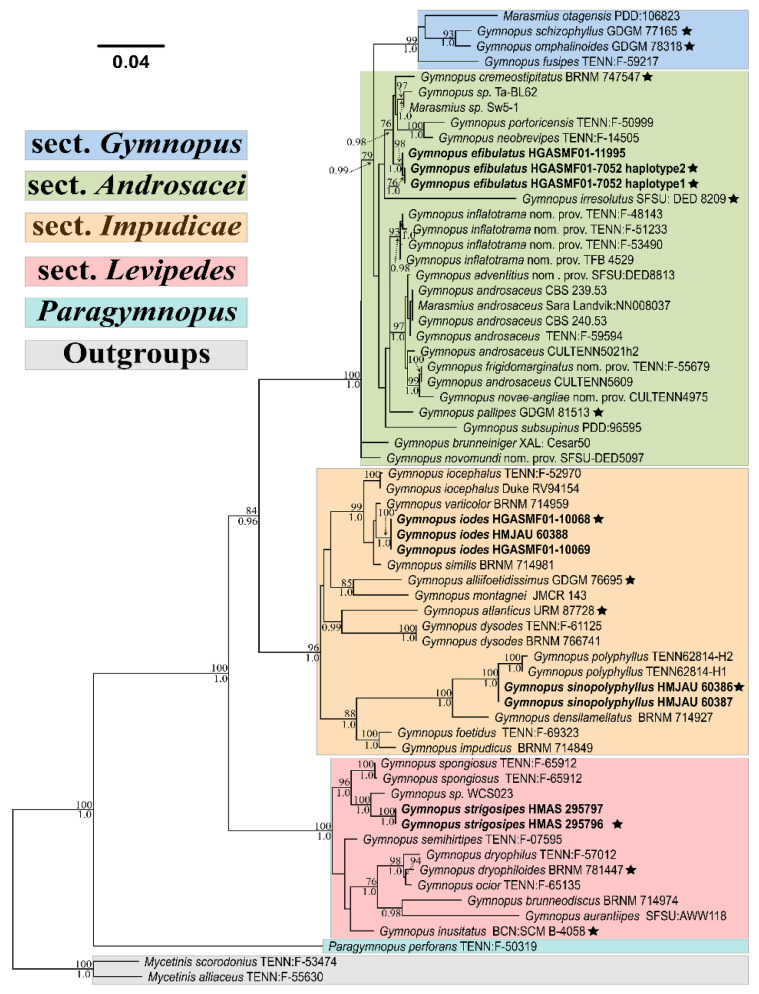
Phylogram inferred from the combined dataset (ITS1-5.8S-ITS2-LSU region) by ML analysis. ML-BP ≥ 70% and BI-PP ≥ 0.95 are shown above and below the branches, respectively. Sequences produced in this study are highlighted in bold, and sequences from type materials are marked with a five-pointed star.

A combined dataset of two markers, including 1712 bases, comprising 64 nrITS sequences and 56 nrLSU sequences, was used to calculate ML and BI analyses. Amongst the dataset, 1237 were constant sites, 128 were variable and parsimony–uninformative sites, and 347 (≈20.27%) were parsimony–informative sites. According to the BIC criterion, the GTR+F+I+G4, K2P, HKY+F+G4 and HKY+F+I+G4 models were selected as the best-fit model for the nrITS1 region, 5.8S marker, nrITS2 region and nrLSU gene, respectively. Only the tree with a better topology generated by the ML method was shown (Figure 1).

In the newly produced phylogram, the clade *Paragymnopus* helps to separate the *Gymnopus* s. str., forming a monophyletic clade with high support (BI-PP/ML-BP = 1.00/100%). Sequences of the nine studied samples were restricted in the *Gymnopus* s. str. clade forming four distinct lineages. Amongst them, the lineage of the collections HGASMF01-11995 and HGASMF01-7052 is highly supported (BI-PP/ML-BP = 1.00/98%) and nested in an unsolved clade representing *G*. sect. *Androsacei*. Moreover, the lineage of the collections HGASMF01-10068, HGASMF01-10069 and HMJAU 60388 is highly supported (BI-PP/ML-BP = 1.00/100%) within a well-supported clade, namely, *G*. sect. *Impudicae* (BI-PP/ML-BP = 1.00/96%). Furthermore, the lineage of the collections HMAS 295796 and HMAS 295797 belongs to the clade of *G*. sect. *Levipedes*, and both are strongly supported by the two methods analyses (BI-PP/ML-BP = 1.00/100%). Besides, collections HMJAU 60386–60387 are clustered in the clade of *G*. sect. *Impudicae*, and their sequences only differ by having two degenerate bases but including the same nucleotide in the corresponding site.

## 4. Taxonomy

**Figure 2:**
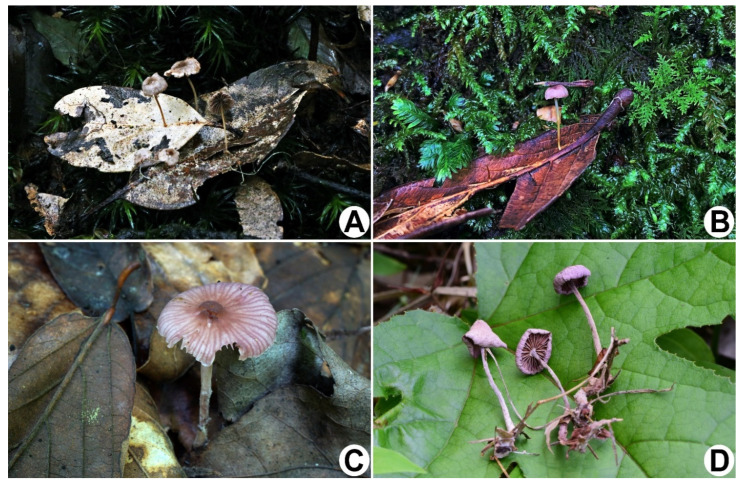
Basidiomata of *Gymnopus*. (**A**,**B**) *G. efibulatus* (**A**) HGASMF01-7052 holotype! (**B**) HGASMF01-11995; (**C**,**D**) *G. iodes* (**C**) HMJAU 60388; (**D**) HGASMF01-10068 holotype!

**Figure 3:**
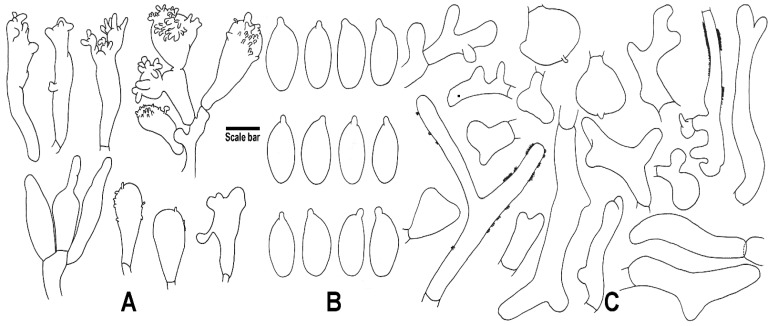
Microscopic features of *G. efibulatus* (HGASMF01-7052, holotype!). (**A**) Cheilocystidia; (**B**) Basidiospores; (**C**) Terminal elements of the pileipellis. Drawing by J.P. Li. Scale bars: 10 μm (**A**,**C**), 5 μm (**B**).

***Gymnopus efibulatus*** J.P. Li, Chang-Tian Li, Chun Y. Deng and Y. Li, sp. nov. (Figure 2A,B and Figure 3).

MycoBank number: MB843239

Etymology: The specific name ‘*efibulatus*’ (Latin), referring to the absence of clamp-connections.

Type: China, Guizhou Province, Tongren City, Fanjingshan National Nature Reserve, 27°54′37″ N, 108°41′53″ E, elev. 2058 m, on dead broadleaves, 28 April 2020, H. Gao, J. Zhang, Z.Q. Shu, D.J. Ou, HGASMF01-7052 (holotype).

Diagnosis: This species is characterized by its brownish grey to dark-brown pileus, whitish to yellowish white rhizomorphs and ellipsoid to oblong basidiospores 7–9 × (3.5–)4–4.5(–5.5) µm in size.

Description: *Basidiomata*: solitary to gregarious. *Pileus*: 5.4–8.8 mm in diam., hemispherical when young, then gradually applanate, shallowly depressed at centre or not, slightly translucently striate towards margin, then sulcate when old, margin entire, then undulating when maturity, dark brown (8F8) overall when very young, gradually changing to paler with age, finally brownish grey (6C2) overall, darker at disc and sulci. *Lamellae*: subdistant, adnate, L = 9–13, l = 1–2, reddish grey (7B2) when young, becoming reddish brown (8D4) when old. *Stipe*: 12.9–15.2 mm long, 0.45–0.6 mm thick at the middle, central and instititious, cylindrical, sometimes compressed at the apex, dry, glabrous, white to whitish at upper part, pastel yellow (1A4) at lower part when young, then almost blonde (4C4) overall, finally becoming cinnamon (6D7) to brown (6E8), more or less paler or darker somewhere. *Rhizomorphs*: present, whitish to yellowish white (1A2), shiny, wiry, simple, repent. *Odour*: negligible.

*Basidiospores*: [*n* = 60] 7–9 × (3.5–)4–4.5(5.5–) µm (average = 7.8 × 4.3 µm, E = (1.51)1.63–2.05(–2.27), Q = 1.85), ellipsoid to oblong, hyaline, thin-walled. *Basidia* [*n* = 20] 19.5–32 × 6–8 µm, clavate, 4-spored. *Basidioles*: [*n* = 20] 20.5–28 × 4.5–8 µm, clavate. *Cheilocystidia* [*n* = 22] 12–40.5 × 5–15 µm, narrowly clavate to broadly clavate, often with more or less finger-like apical projections, sometimes lobed or forming *Siccus*-type broom cells, thin-walled, hyaline. *Pleurocystidia* absent. *Pileipellis*: an entangled, repent cutis of cylindrical, thin-walled, sometimes coarsely incrusted, otherwise smooth hyphae, terminal cells, diverticulate, lobed to irregularly branched, almost coralloid, mixed with some subglobose cells, slightly brownish in KOH. *Stipitipellis*: a cutis composed of cylindrical hyphae, parallelly arranged, often smooth, sometimes with scattered diverticula, sometimes dextrinoid, otherwise inamyloid, slightly thick-walled, hyaline. *Caulocystidia*: absent. *Clamp connections*: absent.

Additional specimen examined: China, Chongqing City, Gold Buddha Mountain National Scenic Area, 29°1′43″ N, 107°11′1″ E, elev. 2098 m, on dead broadleaves, 11 August 2020, J.P. Li, Z.Z. Cen, Q.Y. Lin, M. Wang, HGASMF01-11995.

Notes: Morphologically, the pale-coloured stipe and rhizomorphs of *G. efibulatus* is strongly reminiscent of *G. pallipes* J.P. Li and Chun Y. Deng and *G. cremeostipitatus* Antonín, Ryoo and Ka in the field. However, both *G. pallipes* from China and *G. cremeostipitatus* from South Korea, with smaller basidiospores (6.06 × 3.24 µm, 7.1 × 3 µm resp.) and the latter one with a pubescent stipe and scattered-to-frequent caulocystidia helps distinguish them from the new species [11,17]. Additionally, the absence of clamp connections, which is not quite common in the genus, strongly supports that they are not conspecific [11,17]. What is noteworthy is a description of *Marasmius aurantiobasalis* Desjardin and E. Horak, a member of *Marasmius* sect. *Androsacei* Kühner (≡ *Gymnopus* sect. *Androsacei*), based on individuals from Indonesia shares indistinguishable morphological features with the new species [49]. After checking the original description, *Ma. aurantiobasalis* from New Zealand differs by having smaller basidiospores (6–7.5 × 3–3.5 µm) and a pileipellis of subhymeniform that is made up of clavate to irregular, sometimes lobed, cells with dense diverticula [50].

Phylogenetically, the new species is closely related to *G. cremeostipitatus, G. irresolutus* Desjardin and B.A. Perry, *G. neobrevipes* R.H. Petersen and *G. portoricensis* R.H. Petersen. For the comparison with *G. cremeostipitatus*, see the above paragraph. Additionally, *G. irresolutus* from São Tomé and Príncipe is characterized by a greyish brown to black stipe with minute pruina and the presence of caulocystidia [22]; *G. neobrevipes* from the USA is characterized by the black rhizomorphs and smaller cheilocystidia (2.5–3.5 µm) [8]; *G. portoricensis* from the USA is characterized by the brown to nearly black rhizomorphs, smaller basidiospores [(5–)6–7 × (2.5–)3–4 µm] and the clamped structures [8].

**Figure 4:**
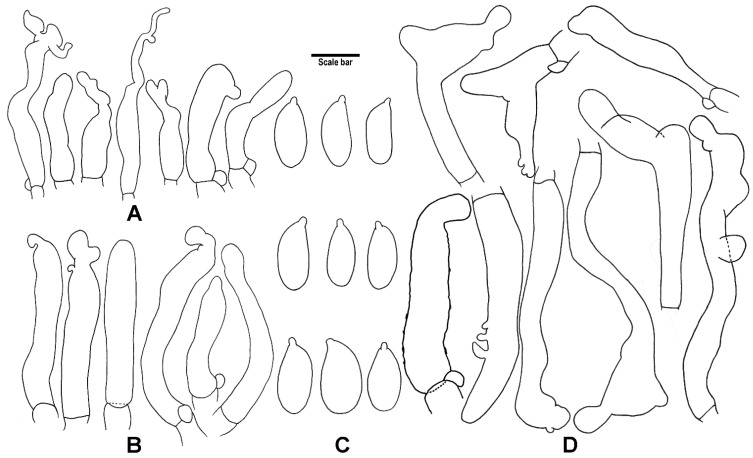
Microscopic features of *G. iodes* (HGASMF01-10068, holotype!). (**A**) Cheilocystidia; (**B**) Caulocystidia; (**C**) Basidiospores; (**D**) Terminal elements of the pileipellis. Drawing by J.P. Li. Scale bars: 10 μm (**A**,**B**,**D**), 5 μm (**C**).

***Gymnopus iodes*** J.P. Li, Chang-Tian Li, Chun Y. Deng and Y. Li, sp. nov. (Figure 2C,D and Figure 4).

MycoBank number: MB843240

Etymology: The specific name ‘*iodes*’ is derived from the ancient Greek, referring to the violet coloured pileus.

Type: China, Guizhou Province, Qiandongnan Miao and Dong Autonomous Prefecture, Liping Country, Deshun village, 26°13′59″ N, 109°22′20″ E, elev. 863 m, on dead twigs or broadleaves, 28 August 2020, J.P. Li, D.F. Wei, M. Wang, HGASMF01-10068 (holotype).

Diagnosis: This species is characterized by its garlicky basidiomata, violet-like pileus and oblong basidiospores 5.5–7.5(–8.5) × 3–4(–4.5) µm in size.

Description: *Basidiomata* solitary. *Pileus* 9.4–21.1 mm in diam., convex to planoconvex, with central papilla or umbo, sometimes with umbilicate centre, when moist radially striate to sulcate-striate except at the centre, then rugulose when drying, crenate margin, when moist greyish red (11D4) to violet brown (11F6) at disc, darker at umbo, fading towards margin to whitish, greyish red (11D5) at sulci, becoming almost pale violet (15A3) overall when drying, darker at sulci. *Lamellae* subdistant, L = 21–27, l = 2–4, emarginate and attached with a very small tooth, adnate when old, rugulose-intervenose at the base, purple grey (13B2), finally becoming greyish magenta (13C3), margin whitish. *Stipe* 16.8–54.2 mm long, 1–1.7 mm thick at the middle, central, cylindrical or laterally compressed, slightly broadened at the apex, entirely whitish tomentose, when young whitish at the upper part, greyish red (10D5) to violet brown (10E5) at the lower part, darker towards the base, almost reddish lilac (11B3) overall when old, with white basal mycelium, dense when old. *Odour*: distinct, garlic-like.

*Basidiospores* [*n* = 60] 5.5–7.5(–8.5) × 3–4(–4.5) µm (average = 6.5 × 3.3 µm, E = (1.67–)1.73–2.30(–2.36), Q = 1.99), oblong, hyaline, thin-walled. *Basidia* [*n* = 20] 18.5–29 × 4.5–6 µm, clavate, 4-spored, hyaline, thin-walled. *Basidioles* [*n* = 20] 21–28 × 5–7.5 µm, hyaline, thin-walled. *Cheilocystidia* [*n* = 30] 13.5–35.5 × 3–6.5 µm, cylindrical or narrowly clavate, irregular, sometimes with forked, rostrate or one irregular filiform apical projection(s), thin-walled. *Pleurocystidia* absent. *Pileipellis* a cutis consisting of interwoven arranged, cylindrical, sometimes slightly incrusted, otherwise smooth hyphae, terminal cells cylindrical to subclavate, irregular to lobate, sometimes with lateral diverticula or projections, turn green in KOH. *Stipitipellis* a cutis composed of cylindrical hyphae, parallelly arranged, hyaline, thin-walled. *Caulocystidia* [*n* = 20] 25.5–75 × 5–9 µm, cylindrical, sometimes with scattered irregular lobate, obtuse apical projections. *Clamp connections* present.

Additional specimens examined: China, Guizhou Province, Qiandongnan Miao and Dong Autonomous Prefecture, Liping Country, Deshun village, 26°13′36″ N, 109°19′44″ E, elev. 842.4 m, on dead broadleaves, 28 August 2020, J.P. Li, D.F. Wei, M. Wang, HGASMF01-10069; Hunan Province, Xiangxi Tujia and Miao Autonomous Prefecture, Yongshun County, Xiaoxi Town, Xiaoxi Village, 28°48′20″ N, 110°15′27″ E, elev. 512 m, on dead broadleaves, 22 July 2021, L.N. Liu, HMJAU 60388.

Notes: Morphologically, *G. iodes* resembles *G. iocephalus* (Berk. and M.A. Curtis) Halling, *G. similis* Antonín, R. Ryoo and K.H. Ka and *G. variicolor* Antonín, Ryoo, Ka and Tomšovský by having a striate pileus, an unpleasant odour and similar-sized basidiospores, which agree to their phylogenetic relationship. However, *G. iocephalus* from the USA, differs by the pileipellis hyphae turning blue in KOH and the hymenium lacking cheilocystidia [6]. Furthermore, *G. similis* from South Korea can be distinguished by the darker (reddish) brown stipe and larger cheilocystidia (20–65 × 5–9 µm) [12], and *G. variicolor* from South Korea, differs by having more brownish pileus, greyish brown or greyish red that becoming pale brownish orange lamellae and larger cheilocystidia (16–40 × 5–9 µm) [12].

**Figure 5:**
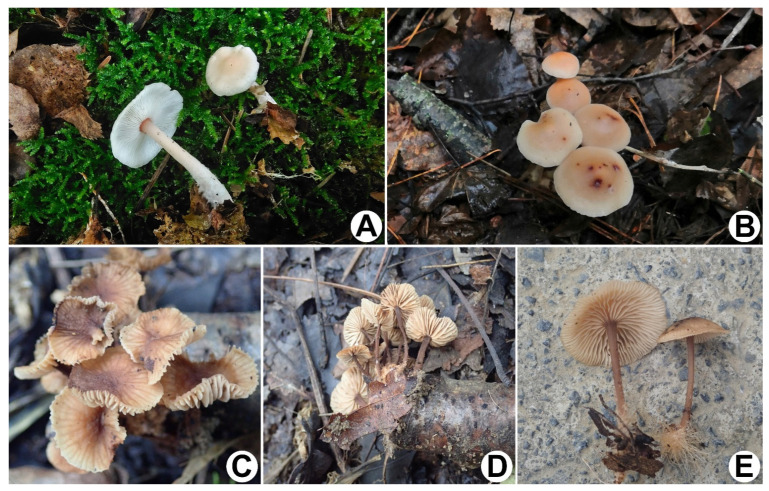
Basidiomata of *Gymnopus*. (**A**,**B**) *G. sinopolyphyllus* (**A**) HMJAU 60387; (**B**) HMJAU 60386 holotype! (**C**–**E**) *G. strigosipes* (**C**,**D**) HMAS 295796 holotype! (**E**) HMAS 295797.

**Figure 6:**
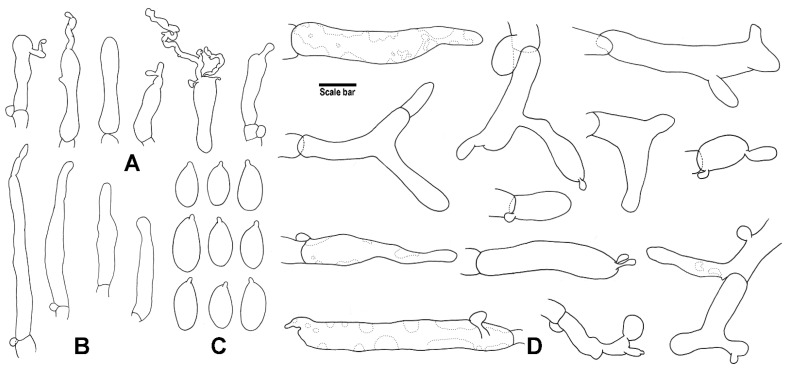
Microscopic features of *G. sinopolyphyllus* (HMJAU 60386, holotype!). (**A**) Cheilocystidia; (**B**) Caulocystidia; (**C**) Basidiospores; (**D**) Terminal elements of the pileipellis. Drawing by J.P. Li. Scale bars: 10 μm (**A**,**B**,**D**), 5 μm (**C**).

***Gymnopus sinopolyphyllus*** J.P. Li, Chang-Tian Li and Y. Li, sp. nov. (Figure 5A,B and Figure 6).

MycoBank number: MB843241

Etymology: The specific name ‘*sinopolyphyllus*’ (Latin), referring to the species described from China and similar to *G. polyphyllus*.

Type: China, Jilin Province, Dunhua City, Xinxing Forest Farm, 43°5′50″ N, 128°10′18″ E, elev. 725 m, on dead broadleaves, 16 April 2021, J.P. Li, N.G. Pan, X. Wang, HMJAU 60386 (holotype).

Diagnosis: This species is characterized by its garlicky basidiomata, reddish-orange to yellowish-grey pileus disc, white powdery-tomentose stipe with basal mycelium, ellipsoid to oblong basidiospores (4.5–)5–7 × (2.5–)3–4 µm in size and pileipellis lacking incrusted hyphae.

Description: *Basidiomata*: solitary to cespitose. *Pileus*: 15–35.5 mm in diam., convex to plano-convex, rounded at disc when young, then expanding to plane, slightly and broadly umbonate at disc, margin entire to slightly uneven, hygrophanous at margin, smooth, glabrous, almost reddish orange (7A6) at disc, gradually fading to orange white (6A2) towards margin, occasionally with dark brown (8F8) tinge on the surface somewhere, finally yellowish grey (3B2) at disc, otherwise whitish. *Lamellae*: very close, free to adnate, very narrow, L = 53–70, l = 4–5, whitish. *Stipe*: 28–58 mm long, 2.5–5 mm thick at the apex, 2.5–5.5 mm thick at the base, cylindrical, slightly broadened at the apex and sometimes also the base, white powdery-tomentose overall, smooth, hollow, whitish to orange white (6A2) tinted with orange red (8B7), darker to garnet brown (9D8) at the apex, sometimes with brownish red (8C8) to reddish brown (8D8) tinge somewhere, with white basal mycelium at the base. *Odour*: distinct, garlic-like.

*Basidiospores*: [*n* = 40] (4.5–)5–7 × (2.5–)3–4 µm (average = 5.9 × 3.2 µm, E = (1.47–)1.58–2.13(–2.3), Q = 1.85), ellipsoid to oblong, hyaline, thin-walled. *Basidia*: [*n* = 30] 20–29.5 × 5–7.5 µm, clavate, 4-spored. *Basidioles*: [*n* = 20] 18.5–28 × 5.5–7 µm, clavate. *Cheilocystidia*: [*n* = 30] 13–43 × 2.5–8.5 µm, narrowly to irregular clavate, smooth, or with one or more projections or irregular and branched outgrowth at the apex, hyaline, thin-walled. *Pleurocystidia*: absent. *Pileipellis*: a cutis consisting of interwoven, cylindrical hyphae, branched, smooth, often with oily contents, sometimes with scattered diverticula, terminal cells cylindrical, sometimes irregularly branched, coralloid at the apex. *Stipitipellis*: a cutis consisting of interwoven, cylindrical hyphae, smooth or with scattered diverticula, thin-walled. *Caulocystidia*: [*n* = 21] 24–62.5 × 3–5.5 µm, cylindrical, often tapering towards the apex. *Clamp connections*: present.

Additional specimens examined: China, Jilin Province, Baishan City, Linjiang City, near Tieshigou Ravine, 41°56′49″ N, 126°44′40″ E, elev. 966 m, on dead broadleaves, 20 July 2021, J.P. Li, N.G. Pan, X. Wang, HMJAU 60387.

Notes: Compared with the species with odorous basidiomata and close to crowded lamellae, *G. atlanticus* V. Coimbra and Wartchow, *G. densilamellatus* Antonín, Ryoo and Ka, *G. hariolorum* (Bull.) Antonín, Halling and Noordel., *G. polyphyllus* (Peck) Halling, and *G. virescens* A.W. Wilson, Desjardin and E. Horak are similar to the new species. However, *G. atlanticus* from Brazil, differs by having a sulcate pileus margin, less lamellulae tiers (l = 3) and smaller basidiospores (7.5 × 3.6 µm) [21]; *G. densilamellatus* from South Korea and *G. polyphyllus* from the USA differ by having incrusted hyphae in pileipellis [6,12]; *G. hariolorum* from Switzerland differs by a more brownish pileus disc and a slightly longitudinally grooved stipe [2]; and *G. virescens* from Indonesia differs by having an entirely dark-brown to dark-reddish-brown stipe, larger basidiospores (7.88 × 3.53 µm) and a pileipellis with brown-incrusted hyphae that turn olivaceous in alkali [15].

Phylogenetic analyses suggest that *G. sinopolyphyllus* is closely affinities with *G. densilamellatus* and *G. polyphyllus*, consistent with the morphological study.

**Figure 7:**
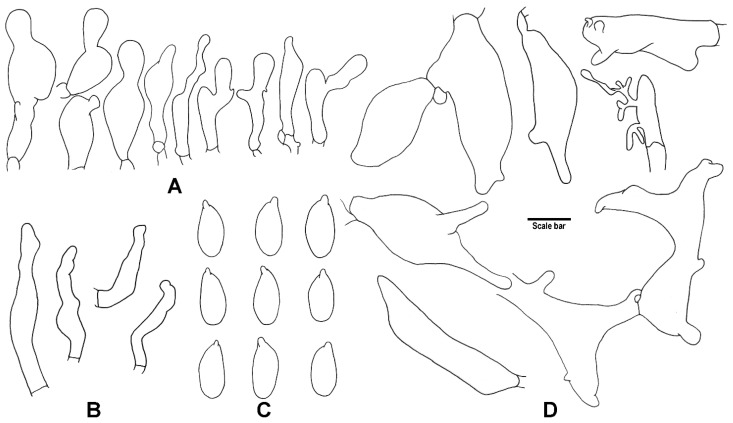
Microscopic features of *G. strigosipes* (HMAS 295796, holotype!). (**A**) Cheilocystidia; (**B**) Caulocystidia; (**C**) Basidiospores; (**D**) Terminal elements of the pileipellis. Drawing by J.P. Li. Scale bars: 10 μm (**A**,**B**,**D**), 5 μm (**C**).

***Gymnopus strigosipes*** J.P. Li, Chang-Tian Li, Yi. Li and Y. Li, sp. nov. (Figure 5C–E and Figure 7).

MycoBank number: MB843242

Etymology: The specific name ‘*strigosipes*’ (Latin), referring to the strigose stipe base.

Type: China, Guizhou Province, Tongren City, Yanhe County, Huangtu town, Huaxi village, Shengjiling Ridge, 28°42′45″ N, 108°16′27″ E, elev. 765 m, on dead broadleaves, 27 October 2020, A. Xu, HMAS 295796 (holotype).

Diagnosis: This species is characterized by its reddish-brown to dark-brown pileus, strigose stipe, oblong basidiospores (5.5–)6–7(–7.5) × 3–3.5(–4) µm in size and the presence of caulocystidia.

Description: *Basidiomata*: cespitose. *Pileus*: 17.5–25 mm in diam., convex to planoconvex when young, then applanate, with rounded to shallowly depressed centre, rugulose at disc when maturity, radially sulcate towards margin, with deflexed to reflexed, finally more or less undulate margin, glabrous, dry, rust brown (6E8) to agate (7E8) at disc when young, gradually becoming reddish brown (8E8) to dark brown (8F8) with age, paler towards margin (nearly orange white (5A2) at margin), but slightly darker at sulci. *Lamellae*: subdistant, adnate, sometimes with a slightly decurrent tooth, arcuate to ventricose, L = 17–21, l = 4–5, orange grey (6B2) to flesh (6B3), whitish at edge. *Stipe* 25–41 mm long, 1.5–2 mm thick at the apex, 1.5–3.5 mm thick at the base, centrally attached, compressed, fibrous, hollow, smooth, tomentose at the lower part, mostly reddish brown overall, more or less paler somewhere, long strigose near the base, whitish to reddish white (7A2). *Odour*: not distinct.

*Basidiospores*: [*n* = 60] (5.5–)6–7(–7.5) × 3–3.5(–4) µm (average = 6.3 × 3.3 µm, E = (1.71–)1.72–2.16(–2.2), Q = 1.94), oblong, hyaline, thin-walled. *Basidia* [*n* = 20] 18–31 × 4.5–6.5 µm, clavate, 4-spored. *Basidioles*: [*n* = 20] 20–32 × 4.5–6.5 µm, clavate. *Cheilocystidia*: [*n* = 50] 13–38.5 × 3.5–13 µm, clavate to narrowly clavate, subfusoid, irregular, lobed, sometimes with filiform apical projection, hyaline, thin-walled. *Pleurocystidia*: absent. *Pileipellis*: a cutis consisting of interwoven, cylindrical hyphae, smooth, terminal cells lobed, irregular branched, coralloid, forming a *Dryophila*-structure. *Stipitipellis*: a cutis composed of cylindrical hyphae, parallelly arranged, hyaline, slightly thick- to thick-walled. *Caulocystidia*: [*n* = 20] 14.5–41 × 3–6 µm, cylindrical, hyaline, thin-walled. *Clamp connections* present.

Additional specimen examined: China, Guizhou Province, Tongren City, Yanhe County, Huangtu town, Huaxi village, Shengjiling Ridge, 28°42′45″ N, 108°16′27″ E, elev. 765 m, on dead broadleaves, 27 October 2020, A. Xu, HMAS 295797.

Notes: Amongst the known species within *G*. sect. *Levipedes* (Quél.) Halling with brownish-coloured pileus and similar lamellae spacing, *G. agricola* Murrill, *G. hybridus* (Kühner and Romagn.) Antonín and Noordel., *G. loiseleurietorum* (M.M. Moser, Gerhold and Tobies) Antonín and Noordel., *G. sepiiconicus* (Corner) A.W. Wilson, Desjardin and E. Horak, *G. spongiosus* (Berk. and M.A. Curtis) Halling and *G. vitellinipes* A.W. Wilson, Desjardin and E. Horak are close to the new species. However, *G. agricola*, from the USA, can be distinguished by its estriate pileus margin, a cartilaginous and non-strigose stipe [5]; *G. hybridus*, from France, and *G. sepiiconicus*, from South Solomons, are characterized by the non-strigose stipe and the absence of caulocystidia [2,14]; *G. vitellinipes*, from Indonesia, has larger basidiospores (8.3–9.3 × 4–4.4 µm) and a poorly developed *Dryophila*-structure in the pileipellis [15]; *G. loiseleurietorum*, from Austria, differs by the absence of true cheilocystidia and hyphae turn green in KOH [2]; *G. spongiosus*, from the USA, has smaller basidiospores (8.4 × 3.6 µm) that often turn olive green in alkali [20].

Phylogenetic analyses agree with the morphological study, which showed the new species is close to *G. spongiosus*.

**Key to species within *Gymnopus* s. str. with morpho-molecular evidence in China**

1. Terminal cells of pileipellis broad, mostly inflated, mixed with irregularly branched elements and some resembling *Dryophila*-type structures ……………………………………………………………………………………………………………………………………………………………………………….. 2.

− Terminal cells of pileipellis coralloid, more or less diverticulate, lobed to irregularly branched, or with *Dryophila*-type structures ……………………………………………………………………………………………………………………………………………………………………………….. 3.

2. Pileus generally deeply umbilicate; lamellae ventricose ………………………………………………………………………………………….... *G. omphalinoides*

− Pileus more or less depressed; lamellae linear to arcuate ………………………………………………………………………………………….... *G. schizophyllus*

3. Rhizomorphs present, cheilocystidia consist of *Siccus*-type broom cells, stipitipellis with dextrinoid hyphae ………………………………………………. 4.

− Rhizomorphs absent, cheilocystidia never a *Siccus*-type broom cell, stipitipellis without dextrinoid hyphae………………………………………………… 5.

4. Clamp connections present ……………………………………………………………………………………………………………………………………. *G. pallipes*

− Clamp connections absent …………………………………………………………………………………………………………………………………….*G. efibulatus*

5. Basidiomata with unpleasant odour …………………………………………………………………………………………………………………………………... 6.

− Basidiomata with negligible odour ……………………………………………………………………………………………………………………………………. 10.

6. Lamellae not close or crowded …………………………………………………………………………………………………………………………………………. 7.

− Lamellae close or crowded ……………………………………………………………………………………………………………………………………………..... 9.

7. Pileus general white overall ………………………………………………………………………………………………………………………... *G. alliifoetidissimus*

− Pileus not white …………………………………………………………………………………………………………………………………………………………… 8.

8. Pileus light brown, orange white to greyish orange when old ……………………………………………………………………………………………. *G. similis*

− Pileus almost reddish lilac overall when drying ………………………………………………………………………………………………………………. *G. iodes*

9. Pileipellis consist of incrusted hyphae ………………………………………………………………………………………………………………. *G. densilamellatus*

− Pileipellis without incrusted hyphae ……………………………………………………………………………………………………………....... *G. sinopolyphyllus*

10. Caulocystidia present ……………………………………………………………………………………………………………………………………... *G. strigosipes*

− Caulocystidia not recorded …………………………………………………………………………………………………………………………………………..... 11.

11. Stipe smooth or tomentose …………………………………………………………………………………………………………………………………………... 12.

− Stipe with hairs …………………………………………………………………………………………………………………………………………………….......... 15.

12. Pileipellis made up of smooth hyphae ……………………………………………………………………………………………………………………………… 13.

− Pileipellis with incrusted hyphae …………………………………………………………………………………………………………………………………….... 14.

13. Basidia sterigmata extremely long, up to 32 µm ……………………………………………………………………………………………………… *G. macrosporus*

− Basidia sterigmata normally long ………………………………………………………………………………………………………………………........... *G. tiliicola*

14. Basidia sterigmata extremely long, up to 33 µm …………………………………………………………………………………………………………… *G. longus*

− Basidia sterigmata normally long ………………………………………………………………………………………………………………………….. *G. globulosus*

15. Pileus tomentose or pileipellis with incrusted hyphae …………………………………………………………………………………………………………… 16.

− Pileus without tomenta and pileipellis made up of smooth hyphae ……………………………………………………………………………………………… 17.

16. Pileus tomentose, pileipellis made up of smooth hyphae …………………………………………………………………………………………… *G. tomentosus*

− Pileus without tomenta, pileipellis with incrusted hyphae …………………………………………………………………………………… *G. longisterigmaticus*

17. Pileus estriate …………………………………………………………………………………………………………………………………………….... *G. erythropus*

− Pileus striate …………………………………………………………………………………………………………………………………………………………....... 18.

18. Stipe longitudinally striate ………………………………………………………………………………………………………………………………....... *G. striatus*

− Stipe smooth ………………………………………………………………………………………………………………………………………………. *G. changbaiensis*

## 5. Discussion

A total of 28 known species of *Gymnopus* s. str. have been reported in China as yet, of which 15 taxa were reported based on morpho-molecular evidence [16,17,18,33]. This study provides descriptions of four new *Gymnopus* species and DNA barcodes. The newly proposed species, except *G. efibulatus*, a member of *G*. sect. *Androsacei*, embrace the current sectional concept well. Four odorous fungi, namely, *G. iocephalus*, *G. iodes*, *G. similis* and *G. variicolor* formed an independent clade implying their close affinities phylogenetically. When revisiting these four gymnopoid fungi in morphology, it is not hard to find that they share the striate pileus and the distant to subdistant lamellae [6,12]. Similarly, *G. densilamellatus*, *G. polyphyllus* and *G. sinopolyphyllus*, forming an independent clade, share the very close to crowded lamellae [6,12]. Besides, *G*. sect. *Androsacei* is still an unsolved clade thus far, phylogenetically. Formally, Li et al. discussed the sectional circumscription and noted the broom cells were absent or weakly present in the pileipellis of several taxa [17]. Furthermore, the pileipellis of *G. efibulatus* also lack the bloom cells but subglobose cells were observed. Accordingly, this section is worthy of further exploration in the future based on morphology inferred from more materials and multilocus phylogenetic analyses.

## Figures and Tables

**Table 1:** Species names, collection numbers and corresponding GenBank accession numbers used in this study.

Species Name	Collection No.	GenBank Accession No		References
		ITS	LSU	
*G. adventitius* nom. prov.	SFSU: DED8813	KY026760	KY026760	[42]
*G. alliifoetidissimus* *	GDGM 76695	MT023348	MT017526	[16]
*G. androsaceus*	CULTENN5609	KY026750	KY026750	[42]
*G. androsaceus*	CBS 240.53	MH857175	MH868714	[43]
*G. androsaceus*	CBS 239.53	MH857174	MH868713	[43]
*G. androsaceus*	CULTENN5021h2	KY026748	KY026748	[42]
*G. androsaceus*	TENN: F-59594	KY026663	KY026663	[42]
*G. atlanticus* *	URM 87728	KT222654	KY302698	[21]
*G. aurantiipes*	SFSU: AWW118	AY263432	AY639410	[15]
*G. brunneiniger*	XAL: Cesar50	MT232388	MW187069	[9]
*G. brunneodiscus*	BRNM 714974	MH589973	MH589988	[10]
*G. cremeostipitatus* *	BRNM 747547	KF251071	KF251091	[11]
*G. densilamellatus*	BRNM 714927	KP336685	KP336694	[12]
*G. dryophiloides* *	BRNM 781447	MH589967	MH589985	[10]
*G. dryophilus*	TENN: F-57012	DQ241781	AY640619	[29]
*G. dysodes*	TENN: F-61125	KY026666	FJ750265	[42]
*G. dysodes*	BRNM 766741	KP336693	KP336701	[12]
***G. efibulatus*** *	**HGASMF01-7052 haplotype1**	**OM970865**	**OM970865**	**This study**
***G. efibulatus*** *	**HGASMF01-7052 haplotype2**	**OM970866**	**OM970866**	**This study**
***G. efibulatus***	**HGASMF01-11995**	**OM970873**	**OM970877**	**This study**
*G. foetidus*	TENN: F-69323	KY026739	KY026739	[42]
*G. frigidomarginatus* nom. prov.	TENN: F-55679	KY026648	KY026648	[42]
*G. fusipes*	TENN: F-59217	AY256710	AY256710	[44]
*G. impudicus*	BRNM 714849	LT594119	LT594119	[12]
*G. inflatotrama* nom. prov.	TENN: F-48143	KY026619	KY026619	[42]
*G. inflatotrama* nom. prov.	TFB 4529	KY026744	KY026744	[42]
*G. inflatotrama* nom. prov.	TENN: F-53490	KY026640	KY026640	[42]
*G. inflatotrama* nom. prov.	TENN: F-51233	KY026632	KY026632	[42]
*G. inusitatus* *	BCN: SCM B-4058	JN247553	JN247557	[3]
*G. iocephalus*	Duke RV94154	DQ449986	unavailable	[7]
*G. iocephalus*	TENN: F-52970	DQ449984	KY019630	[7]
***G. iodes***	**HGASMF01-10069**	**OM970868**	**OM970868**	**This study**
***G. iodes*** *	**HGASMF01-10068**	**OM970869**	**OM970869**	**This study**
***G. iodes***	**HMJAU 60388**	**OM970870**	**OM970870**	**This study**
*G. irresolutus* *	SFSU: DED 8209	MF100973	unavailable	[22]
*G. montagnei*	JMCR 143	DQ449988	AF261327	[7]
*G. neobrevipes*	TENN: F-14505	MH673477	MH673477	[8]
*G. novae-angliae* nom. prov.	CULTENN4975	KY026745	KY026745	[42]
*G. novomundi* nom. prov.	SFSU-DED5097	KY026759	KY026759	[42]
*G. ocior*	TENN: F-65135	KY026678	KY026678	[42]
*G. omphalinoides* *	GDGM 78318	MW134044	MW134730	[18]
*G. pallipes* *	GDGM 81513	MW582856	OK087327	[17,18]
*G. polyphyllus*	TENN62814-H1	FJ596894	unavailable	[45]
*G. polyphyllus*	TENN62814-H2	FJ596895	unavailable	[45]
*G. schizophyllus* *	GDGM 77165	MW134043	MW134729	[18]
*G. semihirtipes*	TENN: F-07595	OK376741	unavailable	GenBank
*G. similis*	BRNM 714981	KP336690	KP336697	[12]
***G. sinopolyphyllus***	**HMJAU 60387**	**OM970871**	**OM970871**	**This study**
***G. sinopolyphyllus*** *	**HMJAU 60386**	**OM970872**	**OM970872**	**This study**
*G.* sp.	WCS023	AB968433	unavailable	[46]
*G.* sp.	Ta-BL62	LC505290	LC505290	[47]
*G. spongiosus*	TENN: F-65912	KY026687	KY026687	[42]
*G. spongiosus*	TENN: F-65912	KY026686	KY026686	[42]
***G. strigosipes*** *	**HMAS 295796**	**OM970874**	**OM970874**	**This study**
***G. strigosipes***	**HMAS 295797**	**OM970867**	**OM970867**	**This study**
*G. subsupinus*	PDD: 96595	KM975399	KM975375	GenBank
*G. variicolor*	BRNM 714959	LT594121	KP348011	[12]
*Ma. androsaceus*	Sara Landvik: NN008037	JN943605	JN941145	[11]
*Ma. otagensis*	PDD: 106823	MT974597	MT974601	[18]
*Ma.* sp.	Sw5-1	LC504952	unavailable	GenBank
*My. alliaceus*	TENN: F-55630	KY696752	KY696752	[48]
*My. scorodonius*	TENN: F-53474	KY696748	KY696748	[48]
*Pa. perforans*	TENN: F-50319	KY026625	KY026625	[42]

Newly generated sequences are highlighted in bold and sequences derived from type specimen are marked with an asterisk (*). The haplotypes were deduced from forward and reverse sequences.

## Data Availability

Publicly available datasets were analyzed in this study. These data can be found here: GenBank, https://www.ncbi.nlm.nih.gov/genbank/ (accessed on 22 March 2022); MycoBank, https://www.mycobank.org/page/Simple%20names%20search (accessed on 22 March 2022); TreeBase, http://purl.org/phylo/treebase, submission ID 29550; (accessed on 18 March 2022). All new taxa were linked with MycoBank (https://www.mycobank.org/ (accessed on 22 March 2022)).
